# Tyrosine Kinase Inhibitor Pazopanib Inhibits Platelet Procoagulant Activity in Renal Cell Carcinoma Patients

**DOI:** 10.3389/fcvm.2018.00142

**Published:** 2018-10-16

**Authors:** Bibian M. E. Tullemans, Magdolna Nagy, Siamack Sabrkhany, Arjan W. Griffioen, Mirjam G. A. oude Egbrink, Maureen Aarts, Johan W. M. Heemskerk, Marijke J. E. Kuijpers

**Affiliations:** ^1^Department of Biochemistry, Cardiovascular Research Institute Maastricht, Maastricht University, Maastricht, Netherlands; ^2^Department of Physiology, Cardiovascular Research Institute Maastricht, Maastricht University, Maastricht, Netherlands; ^3^Angiogenesis Laboratory, Department of Medical Oncology, VU Medical Center, Amsterdam, Netherlands; ^4^Department of Medical Oncology, Maastricht University Medical Center, Maastricht, Netherlands

**Keywords:** platelets, thrombus, tyrosine kinase inhibitor, phosphatidylserine, pazopanib, procoagulant activity

## Abstract

Pazopanib is an angiostatic tyrosine kinase inhibitor (TKI) presently used for cancer treatment, particularly in patients with renal cell carcinoma (RCC). This treatment can be accompanied by mild bleeding as an adverse effect. Given the role of protein tyrosine kinases in platelet activation processes, we investigated whether and how pazopanib can affect platelet functions in purified systems and during treatment of advanced RCC patients. In isolated platelets from healthy volunteers, pazopanib dose-dependently reduced collagen-induced integrin activation and secretion, as well as platelet aggregation. Pazopanib addition diminished glycoprotein (GP) VI-dependent tyrosine phosphorylation of multiple platelet proteins, including the tyrosine kinase Syk. Furthermore, pazopanib inhibited GPVI-induced Ca^2+^ elevation, resulting in reduced exposure of the procoagulant phospholipid phosphatidylserine (PS). Upon perfusion of control blood over a collagen surface, pazopanib inhibited thrombus size as well as PS exposure. Blood samples from 10 RCC patients were also analyzed before and after 14 days of pazopanib treatment as monotherapy. This treatment caused an overall lowering in platelet count, with 3 out of 10 patients experiencing mild bleeding. Platelets isolated from pazopanib-treated patients showed a significant lowering of PS exposure upon activation. In addition, platelet procoagulant activity was inhibited in thrombi formed under flow conditions. Control experiments indicated that higher pazopanib concentrations were required to inhibit GPVI-mediated PS exposure in the presence of plasma. Together, these results indicated that pazopanib suppresses GPVI-induced platelet activation responses in a way partly antagonized by the presence of plasma. In treated cancer patients, pazopanib effects were confined to a reduction in GPVI-dependent PS exposure. Together with the reduced platelet count, this may explain the mild bleeding tendency observed in pazopanib-treated patients.

## Introduction

Tyrosine kinase inhibitors (TKIs) are widely approved drugs, aiming to target tyrosine kinase signaling pathways that regulate uncontrolled cellular growth and proliferation. Currently, several TKIs are in clinical use for the treatment of malignancies, such as lung, breast, kidney, and neuro-endocrine pancreatic cancers as well as gastro-intestinal stromal tumors and chronic myeloid leukemia (1–3). Their common way of action is by competition with adenosine triphosphate (ATP) in the conserved catalytic binding site in the protein tyrosine kinase superfamily. In spite of this action mechanism, individual TKIs can target partially different spectra of intracellular tyrosine kinases, can have different pharmacokinetics, and vary in their adverse effects (1). Commonly, TKIs are clinically applied as a multi-target therapy to intervene in tumor proliferation (4, 5). Specific targets are the receptors for vascular endothelial growth factor (VEGF), platelet-derived growth factor (PDGF) and epidermal growth factor (EGF), which are all involved in (tumor) angiogenesis (3, 6). The expected effects are to reduce tumor lesions, delay disease development, and thus prolong the progression-free survival of patients (7).

Platelets contain several protein tyrosine kinases as key signal transducers, which regulate the function of platelets in hemostasis (8). Downstream of glycoprotein (GP)VI (collagen receptor), GPIb-IX-V (von Willebrand receptor) and CLEC-2 (podoplanin receptor), Src family tyrosine kinases control the signaling routes to most platelet responses (8–10). Activation of GPVI also implies tyrosine phosphorylation of the immunoreceptor tyrosine-based activation motif (ITAM) present on the Fc-receptor γ-chain, which is co-expressed with GPVI (11). This results in activation of the protein tyrosine kinase Syk (10, 12), and further downstream Bruton tyrosine kinase (Btk), culminating in the phosphorylation and activation of phospholipase Cγ2 (PLCγ2), an event required for integrin activation and granule secretion. A similar set of protein tyrosine kinases (Src-family kinases, Syk and JAK isoforms) is known to play a critical role in megakaryocyte development and thrombocytopoiesis (13).

Considering the role of protein tyrosine kinases in both platelet activation and platelet formation, it can be expected that treatment of patients with a broad-spectrum TKI affects hemostasis. Indeed, for several TKIs, anti-hemostatic effects have been reported, like anemia, neutropenia, thrombocytopenia, and bleeding incidences (14–19). In particular, the TKIs sunitinib, ibrutinib, and ponatinib may cause a bleeding risk that relates to impaired GPVI-induced signaling, platelet aggregation, and thrombus formation (14, 15, 18). Also for other TKIs a decrease in platelet count has been described upon treatment (18, 20–22).

Pazopanib is currently used as a first-line therapy for advanced clear-cell renal cell carcinoma (RCC), the most common type of kidney cancer in adults. Pazopanib is aimed to target the VEGF and PDGF receptors, stem-cell factor receptor cKit and Flt-3 (23). The inhibition of these receptors decreases tumor angiogenesis and growth, and hence prolongs patient survival (24, 25). *In vitro* studies have indicated that pazopanib also inhibits several other tyrosine kinase-linked receptors, including fibroblast growth factor receptor, IL-2 receptor inducible T-cell kinase (Itk), leukocyte-specific protein tyrosine kinase (Lck), and the glycoprotein receptor c-Fms (24). In addition, other *in vitro* kinase targets of pazopanib have been described, of which Abl1, Abl2, Fgr, Src, Fyn, and Lck are present in platelets (26, 27). Patients are commonly treated with a high daily doses of pazopanib (800 mg), resulting in a steady-state plasma concentration of up to 45 μg/mL after several weeks (24). This treatment regimen can reduce the platelet count and lead to bleeding events (24). Since effects of pazopanib on platelet function have not been reported, we aimed to investigate this *in vitro* and *ex vivo*, using blood from RCC patients and control subjects.

## Materials and methods

### Materials

Pazopanib (Votrient) was obtained from LC Laboratories (Woburn MA, USA). Arachidonic acid was obtained from Bio/DATA Corporation (Horsham PA, USA), fibrillar Horm type I collagen from Takeda (Hoofddorp, The Netherlands), thrombin from Enzyme Research Laboratories (South Bend IN, USA), thrombin receptor activating peptide 6 (TRAP-6) from Bachem (Bubendorf, Switzerland) and U46619 (thromboxane A_2_ receptor agonist) from Cayman Chemicals (Ann Arbor MI, USA). Fluorescein isothiocyanate (FITC)-labeled PAC-1 monoclonal antibody (mAb) against activated human integrin α_IIb_β_3_ was from BD Bioscience (Franklin Lakes NJ, USA; nr. 340507), while FITC-labeled anti-human CD62 mAb was from Beckman Coulter (Sydney, Australia; nr. 65050). Collagen-related peptide (CRP-XL) was obtained from the University of Cambridge (Cambridge, UK). Methylthio-adenosine-diphosphate (Me-S-ADP), D-phenylalanyl-prolyl-arginyl chloromethyl ketone (PPACK) and mouse anti-Syk antibody were purchased from Santa Cruz Biotechnology (Dallas TX, USA). Fibrinogen and unfractionated heparin were obtained from Sigma-Aldrich (Saint Louis MO, USA). FITC-labeled annexin A5 was from Pharmatarget (Maastricht, The Netherlands). Fura-2-AM was from Invitrogen (Carlsbad CA, USA); Pluronic F-127 from Molecular Probes (Eugene OR, USA). For Western blotting, the following antibodies were used: mouse anti-phosphotyrosine mAb (clone 4G10) obtained from Millipore (Billerica MA, USA), rabbit anti-tubulin Ab from Abcam (Cambridge, UK), rabbit anti-phospho Syk T525/526, HRP-conjugated anti-rabbit-IgG from Cell Signaling, (Leiden, The Netherlands) and HRP-conjugated anti-mouse-IgG from VWR international (Amsterdam, The Netherlands).

### Blood collection from patients and healthy volunteers

The study was approved by the medical ethics committee of the Maastricht University Medical Center^+^ (MUMC^+^, The Netherlands). All participants provided written informed consent in accordance with the Declaration of Helsinki. Blood was obtained from 35 healthy volunteers and ten patients diagnosed with metastatic RCC at the Department of Medical Oncology of MUMC^+^. Patients were included, if eligible for treatment with pazopanib as a single agent (800 mg/day). Excluded were subjects who used anticoagulants or platelet inhibitory drugs. From healthy volunteers, one blood sample was collected, while the patients donated two blood samples: 1 day before and at 14 days after starting pazopanib treatment, i.e., when a steady-state plasma concentration was reached (24). Blood samples were collected from the antecubital vein into 3.2% trisodium citrate; the first 5 mL of blood was discarded after which 10 mL was collected.

### Blood composition and platelet isolation

Hematological parameters, including platelet count, were determined with a Sysmex XP300 (Chuo-ku Kobe, Japan). Washed platelets were obtained as described (28). In brief, platelet-rich plasma (PRP) was collected after 15 min centrifugation at 240 *g*, followed by a washing step. Washed platelets were resuspended into Hepes buffer pH 7.45 [10 mM Hepes, 136 mM NaCl, 2.7 mM KCl, 2 mM MgCl_2_, 0.1% glucose, and 0.1% bovine serum albumin (BSA)]. Platelet count was adjusted, as required for the particular assay.

Blood from healthy volunteers was used for *in vitro* experiments of pazopanib effects. Samples of whole blood, PRP or washed platelets were pre-incubated with pazopanib or vehicle (dimethylsulfoxide) for 10 min at 37°C.

### Light transmission aggregometry

Aggregation of platelets, washed or in PRP (250 × 10^9^ platelets/L) was measured using a Chronolog aggregometer (Havertown PA, USA) under constant stirring (37°C); pazopanib (5, 10, or 30 μM) or vehicle was present as indicated. Aggregation responses were quantified as maximal amplitude in light transmission (29). Aggregation of washed platelets was induced with collagen (1 μg/mL), Me-S-ADP (1 μM) in the presence of fibrinogen (25 μg/mL), thrombin (1 nM), TRAP-6 (10 μM), U46619 (1 μM), or arachidonic acid (10 μM). Aggregation of platelets in PRP was induced with collagen (1 μg/mL).

### Flow cytometry

Washed platelets (100 × 10^9^ platelets/L) were incubated for 10 min at 37°C with vehicle or pazopanib (10 μM). The cells in Hepes buffer pH 7.45 containing 2 mM CaCl_2_ were then stimulated with CRP-XL (1 μg/mL), Me-S-ADP (1 μM), or thrombin (1 nM). Using described flow cytometry procedures, integrin α_IIb_β_3_ activation and P-selectin expression were determined with FITC-conjugated PAC1 mAb (1:10) and FITC-conjugated anti-CD62P mAb (1:10), respectively (30). For the measurement of PS exposure, platelets were stimulated with CRP-XL (5 μg/mL) and thrombin (4 nM) for 60 min at 37°C (31). Exposure of PS was determined with FITC-conjugated annexin A5 (1 μg/mL).

To assess PS exposure in the presence of plasma, platelet count of washed platelets or PRP were adjusted to 100 × 10^9^/L. Washed platelet were diluted in Hepes buffer pH 7.45 with 2 mM CaCl_2_; PRP was diluted with autologous plasma supplemented and 6.3 mM CaCl_2_ plus 3.2 mM MgCl_2_. Washed platelets and PRP were mixed in various ratios to obtain 0, 10, 30, 50, and 100% plasma. The mixed samples were preincubated with pazopanib (1, 5, 10, 30, 50, 75, or 100 μM) or vehicle for 10 min at 37°C, and then activated with CRP-XL (5 μg/mL) and TRAP-6 (15 μM) in the presence of PPACK (40 μM). Exposure of PS was determined after 60 min at 37°C with FITC-conjugated annexin A5 (1 μg/mL). Flow cytometric measurements were performed in duplicate using a BD Accuri C6 flow cytometer and corresponding software (Erembodegem, Belgium).

### Whole blood perfusion experiments

Whole blood perfusion experiments were performed as described before (32). In short, citrate-anticoagulated blood samples were incubated with pazopanib (30 μM) or vehicle for 10 min at room temperature. After recalcification in the presence of thrombin inhibitor (40 μM PPACK, 6.3 mM CaCl_2_, 3.2 mM MgCl_2_, f.c.), the samples were perfused through a transparent parallel-plate flow chamber, containing a coverslip coated with type I collagen (50 μg/mL) at a wall-shear rate of 1,000 s^−1^. After 4 min, thrombi formed on coverslip were stained with FITC-conjugated annexin A5 (1 μg/mL in Hepes buffer pH 7.45, containing 2 mM CaCl_2_ and 1 U/mL heparin). At least 10 random brightfield and fluorescence images were captured with an EVOS microscope (Bothell WA, USA). Microscopic digital images were analyzed for platelet deposition (% of surface area coverage, SAC), multilayer % SAC, integrated feature size and staining for PS (% SAC), using ImageJ 1.45s software (ImageJ ecosystem, from imagej.nih.gov/ij/). The integrated feature size is a parameter of platelet aggregation, taking into account the proportional contribution of large and small thrombi on microspots (33). Further details of image analysis are described elsewhere (34).

### Cytosolic Ca^2+^ measurements

Washed platelets were incubated with Fura-2-AM (3 μM) in the presence of pluronic (600 μM) for 45 min at 37°C. After another wash step, the Fura-2 loaded platelets (100 × 10^9^/L) were used for cytosolic Ca^2+^ ([Ca^2+^]_i_) measurements, as described previously (35). In brief, using polystyrene cuvettes, platelets in suspension (0.5 mL) were pre-incubated with pazopanib (10 μM) or vehicle for 8 min at room temperature and 2 min at 37°C. After baseline measurement, CaCl_2_ (1 mM) was added, followed by an agonist. Changes in Fura-2 fluorescence were measured by ratio fluorometry at dual excitation wavelengths of 340 and 380 nm and an emission wavelength of 510 nm. After correction for background fluorescence, ratio values were converted into levels of [Ca^2+^]_i_. Maximal rises in [Ca^2+^]_i_ and [Ca^2+^]_i_-time integrals (4 min) were determined (36).

### Western blotting

Washed platelets (500×10^9^/L) were pre-incubated with pazopanib (10 or 30 μM) or vehicle and stimulated under stirring conditions with CRP-XL (5 μg/mL) or left unstimulated. Samples were lysed with NP40 lysis buffer, supplemented with a cocktail of phosphatase inhibitors, and protein content was determined using a BioRad protein assay (Hercules CA, USA). Platelet lysates were separated by polyacrylamide gel electrophoresis and subjected to standard western blotting. Blots were stained for tyrosine phosphorylation profile as described (18), using anti-phosphotyrosine mAb 4G10 (1:2,000) and secondary HRP-conjugated Ab (1:500). Specific phosphorylation of Syk was visualized, as described (37), using anti-Syk Tyr^525/526^ mAb (1:1,000) and secondary HRP-conjugated secondary Ab (1:500). Total Syk was determined by reprobing with anti-Syk mAb (1:1,000) and HRP-conjugated secondary Ab (1:1,000). As a control for total platelet proteins, blots were also probed for α-tubulin (1:1,000). The intensity of stained bands was analyzed with ImageJ 1.45s software.

### Statistical analysis

Data were checked for Gaussian distribution using the Kolmogorov-Smirnov Normality test. Normally distributed data are presented as means ± SEM, whereas data with skewed distribution are presented as median ± interquartile range. Statistical significance between *in vitro* data-sets (vehicle vs. pazopanib-treated samples) was determined using paired *t*-test. Paired data-sets of the patients (before and after pazopanib therapy) were compared using the Wilcoxon matched-pairs signed-rank test. When more than 2 conditions were compared, a one-way ANOVA was used. GraphPad Prism 5.0 software (La Jolla CA, USA) was used for statistical analyses. A *P* < 0.05 was considered to be statistically significant.

## Results

### Pazopanib *in vitro* suppresses collagen- and ADP-induced platelet aggregation, secretion, and PS exposure

**Figure 1:**
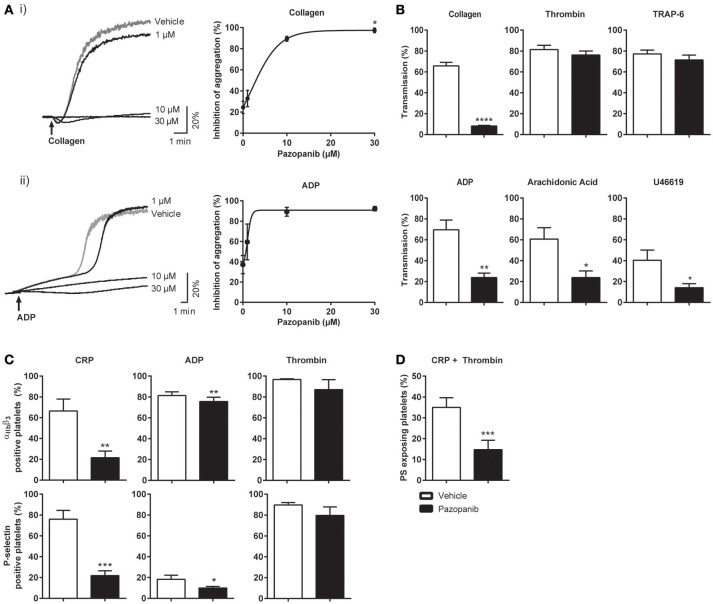
Pazopanib inhibits collagen- and ADP-induced platelet aggregation, integrin activation, secretion and PS exposure. Washed platelets from healthy donors were incubated with 1, 10, or 30 μM pazopanib or vehicle (0.1% DMSO) for 10 min. **(A)** Representative aggregation curves and dose-response graphs of inhibition of aggregation (*n* = 4). Arrow indicates addition of (i) 1 μg/mL collagen or (ii) 25 μg/mL fibrinogen and 1 μM Me-S-ADP. **(B)** Aggregation of washed platelets in presence (black bars) or absence (white bars) of 10 μM pazopanib was induced by 1 μg/mL collagen, 1 nM thrombin, 10 μM TRAP-6, 1 μM Me-S-ADP in presence of 25 μg/mL fibrinogen, 30 μM arachidonic acid or 1 μM U46619. Histograms indicate maximal amplitude of aggregation (*n* = 6). **(C)** Platelets, pre-treated with vehicle or pazopanib (10 μM), were stimulated with 5 μg/mL CRP-XL, 1 μM Me-S-ADP or 1 nM thrombin for 10–20 min, and analyzed by flow cytometry (*n* = 6). Shown are percentages of platelets binding FITC-labeled PAC-1 monoclonal antibody against integrin α_IIb_β_3_ or FITC-labeled anti-human CD62 mAb. **(D)** Platelets pre-treated with vehicle or pazopanib (10 μM) were stimulated with 5 μg/mL CRP-XL and 4 nM thrombin for 60 min, and analyzed by flow cytometry (*n* = 6). Exposure of PS was determined by FITC-labeled annexin A5. Histogram shows percentages of platelets binding FITC-annexin A5. Data are means ± SEM, ^*^p < 0.05, ^**^p < 0.01, ^***^p < 0.001, ^****^p < 0.0001.

Platelet activation in hemostasis involves multiple agonists and their receptors (38). Adhesion of platelets to extracellular matrix proteins, like collagen, is followed by platelet activation (characterized by integrin α_IIb_β_3_ activation and secretion), aggregation (via released ADP and thromboxane A_2_) and thrombus formation. Highly activated platelets also expose the procoagulant phospholipid PS, which promotes thrombin generation and fibrin formation (39, 40). We first investigated the effect of pazopanib on the aggregation response of washed platelets to collagen (acting via GPVI) or ADP (acting via P2Y receptors). Dose-response curves indicated near-complete inhibition with either agonist already at a relatively low dose of 10 μM pazopanib (Figure 1A). In following experiments, the concentration of 10 μM appeared to suppress platelet aggregation with ADP, arachidonic acid and U46619 (thromboxane A_2_ analog). On the other hand, platelet aggregation induced by thrombin or the PAR-1 receptor agonist, TRAP-6, was not affected by pazopanib at this dose (Figure 1B).

Subsequently, flow cytometry was used to determine the effects of pazopanib on specific platelet responses, i.e., integrin α_IIb_β_3_ activation and α-granule secretion (P-selectin expression). Markedly, pazopanib at 10 μM strongly inhibited integrin activation and granule secretion induced by the GPVI receptor agonist (CRP-XL), with no or limited effect in response to ADP or thrombin stimulation (Figure 1C). Pazopanib also reduced CRP-XL plus thrombin-induced PS exposure by >50% (Figure 1D). Taken together, these results indicate that, in washed platelets, pazopanib is an efficient antagonist of platelet responses induced by GPVI agonists (collagen or CRP-XL), but is less effective in antagonizing responses induced by the G-protein coupled receptor agonists ADP, thromboxane A_2_ or thrombin.

### Pazopanib inhibits phosphorylation of Syk, and reduces platelet Ca^2+^ responses

**Figure 2:**
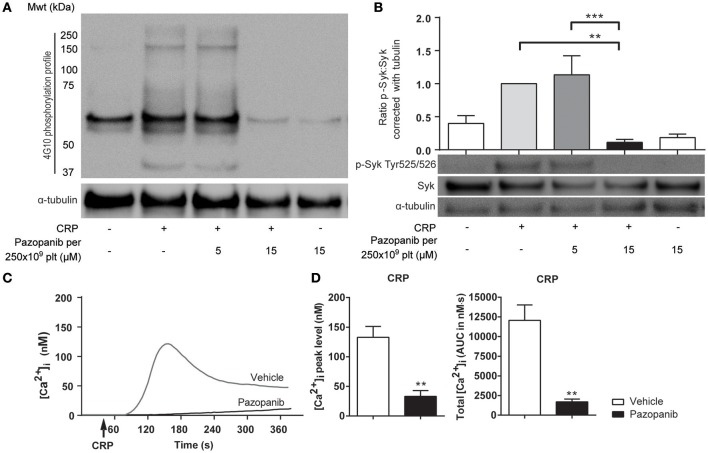
Pazopanib inhibits GPVI-induced protein tyrosine phosphorylation via Syk and cytosolic Ca^2+^ rises. **(A,B)** Representative western blots (*n* = 4) with washed platelets (500 × 10^9^/L) incubated with vehicle or pazopanib (10 or 30 μM) for 10 min, and stimulated with 5 μg/mL CRP-XL for 3 min. For comparison to Figure 1, pazopanib concentrations were re-calculated to match a platelet concentration of 250 × 10^9^/L. **(A)** Protein tyrosine phosphorylation was visualized with 4G10 mAb. Total amount of protein was visualized by staining for α-tubulin. **(B)** Protein phosphorylation of Syk (Tyr^525/526^) and Syk, and parallel staining for α-tubulin. Shown is the ratio of phospho-Syk/total Syk assessed by gray intensity analysis (*n* = 4). **(C)** Representative traces of changes in cytosolic Ca^2+^ of Fura-2-loaded platelets (100 × 10^9^/L), pre-incubated with vehicle or 10 μM pazopanib for 10 min, and stimulated with 1 μg/mL CRP-XL or 1 nM thrombin in the presence of 1 mM CaCl_2_. **(D)** Histograms show maximal rise in [Ca^2+^]_i_ and total [Ca^2+^]_i_ (area-under-curve, AUC) (*n* = 5). Data are means ± SEM, ^**^p < 0.01, ^***^p < 0.001.

The decreased platelet responses to GPVI agonists suggested that pazopanib influences platelet signaling via the tyrosine kinase-operating ITAM pathway. To investigate this in more detail, we assessed the effect of pazopanib on protein tyrosine phosphorylation patterns of GPVI-stimulated platelets using western blotting. Pre-incubation of 500×10^9^ platelets/L with 30 μM pazopanib (i.e., equivalent to 15 μM for 250 × 10^9^ platelets/L, used for aggregation experiments) strongly reduced the CRP-XL induced tyrosine phosphorylation of multiple proteins (Figure 2A). This suggested interference of pazopanib early in the GPVI signaling cascade. This was confirmed by the finding that pazopanib strongly inhibited the phosphorylation of Syk (Figure 2B).

GPVI signaling via Syk results in elevation of intracellular Ca^2+^ levels ([Ca^2+^]_i_) as a prerequisite for integrin activation, secretion and PS exposure. To investigate this, platelets were loaded with the Ca^2+^ probe Fura-2, and agonist-induced responses were measured. In platelets stimulated with CRP-XL, the presence of pazopanib resulted in a reduction of the maximal (peak height) and total (area-under-curve) Ca^2+^ rises (Figures 2C,D). Pazopanib did however not influence [Ca^2+^]_i_ elevation in response to thrombin stimulation (not shown). These results confirm that pazopanib interfered in the early GPVI-induced signaling cascade.

### Pazopanib affects thrombus size and PS exposure in whole blood under flow

**Figure 3:**
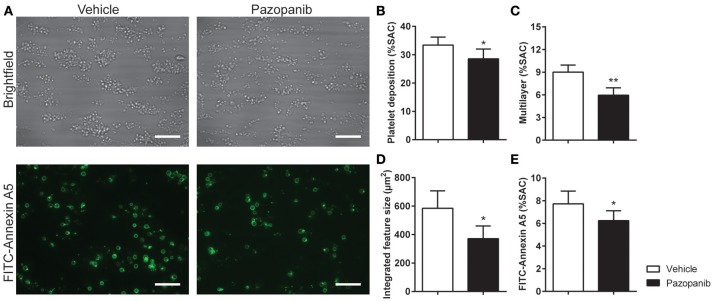
Pazopanib suppresses thrombus formation in whole blood perfused over collagen. Whole blood from healthy volunteers was perfused for 4 min at wall shear rate of 1,000 s^−1^ over microspots containing collagen-I. **(A)** Representative brightfield images (upper panel) and fluorescence images of PS exposure (lower panel) of control blood sample incubated with 30 μM pazopanib. Bar = 20 μm. **(B–E)** Quantification (*n* = 6) of brightfield images of: platelet deposition **(B)**, multilayered thrombus **(C)**, cumulative size of thrombus (integrated feature size) **(D)**, and fluorescence images of staining for PS exposure **(E)**. Data are means ± SEM (*n* = 6), ^*^p < 0.05, ^**^p < 0.01.

Platelet activation via GPVI is an essential step in collagen-induced thrombus formation in whole blood under flow conditions (41). This method was used to determine the effect of pazopanib on platelets in a whole blood environment. Pre-incubation of blood with 30 μM pazopanib resulted in a small, but significant decrease in platelet deposition compared to vehicle-treated blood (Figures 3A,B). Furthermore, the height of thrombi with multilayered platelets was reduced with pazopanib (Figure 3C). The aggregate-reducing effect was confirmed by measuring the size distribution of thrombi (integrated feature size), which was significantly decreased after treatment of the blood with pazopanib (Figure 3D). Post-staining with fluorescently labeled annexin A5 indicated a lower PS exposure (Figure 3E), as in agreement with the flow cytometric results using washed platelets.

### Pazopanib treatment of renal cell carcinoma patients moderately affects platelet functions

**Figure 4:**
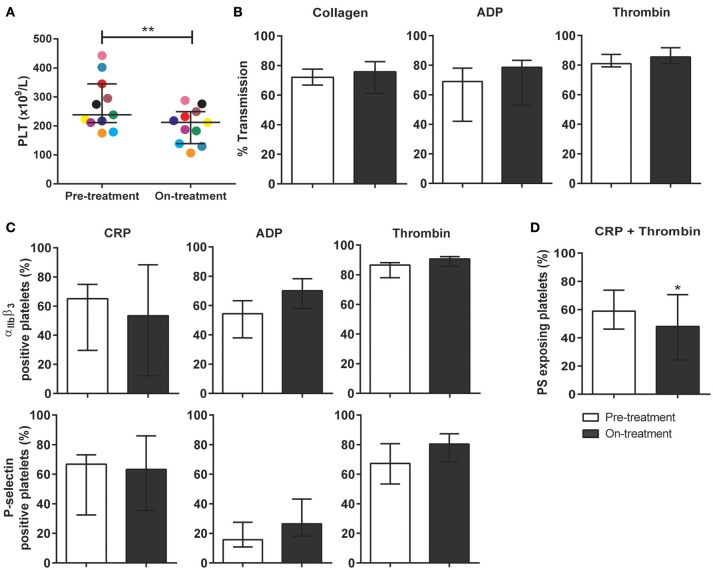
Treatment of carcinoma patients with pazopanib decreased platelet count and suppressed phosphatidylserine exposure in washed platelets. Blood from patients was collected the day before and at 2 weeks after pazopanib treatment. **(A)** Platelet count was measured in blood from RCC patients before and on treatment with pazopanib. Each dot represents a single patient. **(B)** Platelets were isolated and aggregation was induced by 1 μg/mL collagen, 1 μM Me-S-ADP (in the presence of 25 μg/mL fibrinogen), or 1 nM thrombin. Histograms indicate maximal amplitude of aggregation (*n* = 6). **(C)** Platelets before and during treatment of patients were stimulated with 5 μg/mL CRP-XL, 1 μM Me-S-ADP or 1 nM thrombin for 10–20 min, and analyzed by flow cytometry (*n* = 10). Shown are percentages of platelets binding FITC-PAC1 mAb (α_IIb_β_3_ expression) or FITC-anti-CD62P mAb (P-selectin expression). **(D)** Patient platelets were stimulated with 5 μg/mL CRP-XL and 4 nM thrombin for 60 min, and analyzed by flow cytometry (*n* = 6). Histogram shows percentages of platelets binding FITC-annexin A5 (PS exposure). Data are medians ± interquartile ranges, ^*^p < 0.05, ^**^p < 0.01.

To investigate the clinical relevance of these findings, blood samples were obtained from 10 patients diagnosed with metastatic RCC, and eligible for pazopanib treatment (Table 1). The patients (6 females) had a mean age of 69 (range: 51–88) years. Blood samples were collected at 1 day prior and at 14 days after the start of pazopanib treatment. Median platelet count in the patients' blood before start of treatment was 230 × 10^9^/L (interquartile range: 213–290 × 10^9^/L), while this moderately, but significantly reduced to 201 × 10^9^/L (interquartile range: 150–241 × 10^9^/L) after treatment (Figure 4A). Bleeding complications were reported for three patients, who all developed mild epistaxis (Table 1).

**Table 1:** Characteristics of patients treated with pazopanib 800 mg/day.

**Patient no**.	**Age range**	**Platelet count**	**Platelet count**	**Bleeding**
	**(years)**	**day 0 (x10^9^/L)**	**day 14 (x10^9^/L)**	
1	56–60	238	183	No
2	61–65	222	212	No
3	71–75	175	107	Yes, epistaxis
4	61–65	212	189	No
5	66–70	217	219	Yes, epistaxis
6	81–85	443	288	Yes, epistaxis
7	66–70	402	130	No
8	51–55	295	249	No
9	76–80	274	276	No
10	81–85	179	139	No

*Metastatic renal cell carcinoma patients were included, if eligible for treatment with pazopanib as a single agent. The patients (6 females) had a mean age of 69 (range: 51–88) years. The patients donated two blood samples: 1 day before (day 0) and at 14 days after starting pazopanib treatment*.

Using washed platelets from patients before and after pazopanib treatment, aggregation was determined by light transmission aggregometry. Strikingly, no treatment effect could be observed upon collagen stimulation (Figure 4B). Flow cytometry was used to assess α_IIb_β_3_ integrin activation and secretion upon stimulation with CRP-XL, ADP or thrombin. Again, no effect of the treatment on these platelet responses could be observed (Figure 4C). On the other hand, platelet procoagulant activity, as determined from PS exposure in response to CRP-XL plus thrombin, was significantly decreased by 18% after treatment (Figure 4D).

**Figure 5:**
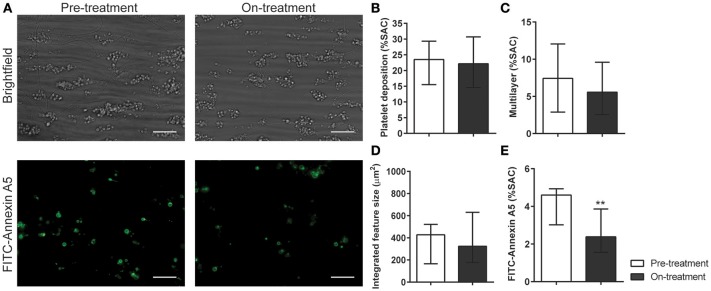
Treatment of pazopanib patients suppressed phosphatidylserine exposure upon thrombus formation on collagen. **(A)** Whole blood from patients was perfused for 4 min at wall shear rate of 1,000 s^−1^ over a collagen surface. Shown are representative brightfield images (upper panel) and fluorescence images of PS exposure (lower panel) for blood samples taken before and after 2 weeks of pazopanib treatment. Bar = 20 μm. **(B–E)** Quantification of brightfield images of platelet deposition **(B)**, multilayered thrombus **(C)**, cumulative size of thrombus (integrated feature size, IFS) **(D)**, and fluorescence images of staining for PS exposure **(E)**. Data are medians ± interquartile ranges (*n* = 10), ^**^p < 0.01.

In whole blood flow experiments over collagen, parameters of thrombus formation were investigated before and after pazopanib treatment. Whereas, platelet deposition and thrombus buildup were not affected in the post-treatment blood samples (Figures 5A–D), a significant reduction of 48% was seen in PS exposure in the post-treatment samples, as compared to pre-treatment (Figures 5A,E).

### Plasma impairs pazopanib effects on platelet function

**Figure 6:**
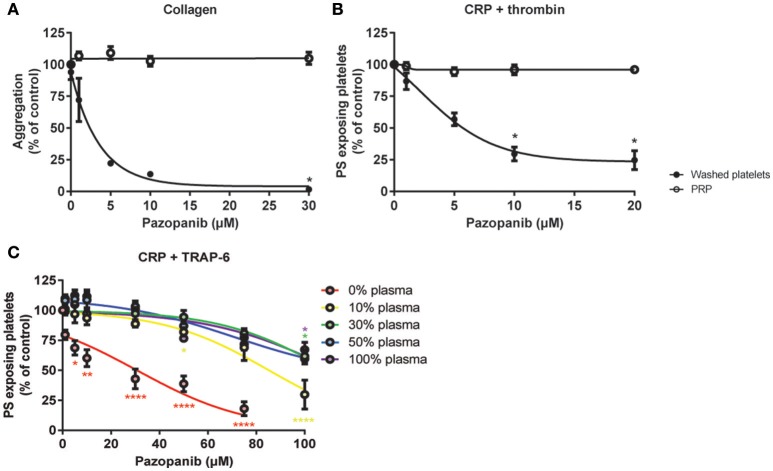
Plasma suppresses effects of pazopanib on platelet activation *in vitro*. Washed platelets or PRP from healthy donors were incubated with 1, 5, 10, or 30 μM pazopanib or vehicle (0.1% DMSO) for 10 min. **(A)** Aggregation of platelets in buffer or PRP in response to 1 μg/mL collagen. **(B)** Exposure of PS measured in washed platelets or in PRP, stimulated with 5 μg/mL CRP-XL and 4 nM thrombin for 60 min, and analyzed by flow cytometry using FITC-labeled annexin A5. **(C)** Platelets reconstituted at different plasma concentrations were pre-incubated with pazopanib (1–100 μM), and stimulated with 5 μg/mL CRP-XL plus 15 μM TRAP-6; PS exposure was quantified by flow cytometry using FITC-labeled annexin A5 (PS exposure). Data are means ± SEM (*n* = 3), ^*^p < 0.05, ^**^p < 0.01, ^****^p < 0.0001.

In isolated platelets we observed a dose-dependent inhibiting effect of pazopanib on platelet function via the inhibition of tyrosine phosphorylation of Syk, and downstream intracellular Ca^2+^ signaling. This was accompanied by reduced integrin activation, secretion and PS exposure. In contrast, pazopanib treatment of RCC patients only resulted in reduction of platelet PS exposure. We hypothesized that the presence of plasma can interfere with the antiplatelet activity of pazopanib. To investigate this, experiments were performed with both PRP and washed platelets, which were incubated with different concentrations of pazopanib. In undiluted PRP, collagen-induced aggregation was not affected by pazopanib, whereas this response was strongly inhibited at 10 μM in washed platelets (Figure 6A). Pre-incubation of PRP with increasing doses of pazopanib until 20 μM did not inhibit PS exposure, in contrast to pre-incubation of washed platelets (Figure 6B).

It has been described that pazopanib is highly bound by plasma proteins at concentration ranges of 10–100 μg/mL (24). This corresponds with a concentration of 23–230 μM. Hence, also higher concentrations of pazopanib (50–100 μM) were tested in platelets incubated at different plasma concentrations (0, 10, 30, 50, or 100% plasma). It appeared that pazopanib treatment substantially suppressed PS exposure in response to CRP-XL plus TRAP-6 in the presence of low amounts of plasma (Figure 6C). However, at 100 μM pazopanib, PS exposure was still significantly reduced by 25% with 30–100% plasma (Figure 6C, *p* < 0.01). This effect is comparable to the moderate inhibition of PS exposure in patients treated with pazopanib (see Figure 4D). These results suggest that the presence of plasma interferes with the incorporation of pazopanib into platelets.

## Discussion

In the present study, we demonstrate that the multi-target TKI pazopanib markedly reduces the collagen-induced activation responses of isolated platelets, including aggregation, PS exposure and Ca^2+^ signaling through inhibition of tyrosine kinases, including Syk. In blood samples from RCC patients treated with pazopanib, these effects were confined to a suppression of GPVI-mediated PS exposure, observed in isolated platelets as well as in whole blood thrombus formation under flow.

Several protein tyrosine kinases are known to contribute to platelet activation processes and hemostasis (8, 10, 11). An activity-based kinase profiling already showed that pazopanib can target various tyrosine kinases that are highly expressed in platelets (42). Here we confirm that, in washed platelets, the GPVI-dependent phosphorylation of multiple proteins, including Syk, is suppressed by pazopanib, in a way accompanied by reduced platelet activation processes. Syk is known to have multiple sites of phosphorylation which both regulate activity and serve as docking motifs for other proteins (43). These sites include Tyr-348 and Tyr-352 within the SH2-linker region, Tyr-525 and Tyr-526 within the activation loop of the kinase domain, Tyr-630 in the C terminus of Syk, and other sites such as Thr-384 and Ser-297. It has been shown that Tyr-525/526 is essential for Syk function (44). Therefore, we selected this phosphorylation site to study the effect of pazopanib on platelet function. However, from the present data it cannot be concluded whether pazopanib inhibits Syk directly, or if its phosphorylation is reduced through inhibition of upstream tyrosine kinases. Most likely, it can be both as pazopanib has been shown to have affinity for multiple TKs in platelets. We have observed that the Syk inhibitor II completely inhibits PS-exposure in washed platelets (37), which was much stronger than the inhibitory effect of pazopanib (Figure 1D). Furthermore, deficiency and selective inhibition of Syk has been shown to prevent platelet aggregation and activation in response to collagen-receptor stimulation in mice (45).

Interestingly, pazopanib also suppressed platelet responses to ADP, arachidonic acid and TxA_2_ analog, while responses to thrombin were unaffected. In agreement with this is the recent finding that three tyrosine kinases are phosphorylated upon platelet activation with ADP using phosphoproteomics (46). These are JAK3, BTK, and TNK2, indicating TKs are involved in signaling underneath ADP. It should be noted that these TKs are phosphorylated on residues other than tyrosines, and the functional consequences are not known. Whether TKs are also phosphorylated under arachidonic acid and thromboxane A_2_ receptor stimulation has not yet been investigated with phosphoproteomics, but this is not unlikely. Although it appears that the majority of the targets of pazopanib are underneath GPVI, these data indicate that pazopanib may also target kinase events downstream of other platelet receptors. Moreover, as it has been shown that inhibition of GPVI alone prevents occlusive thrombus formation without causing bleeding (47), these results support the hypothesis that the targeting of pathways downstream of additional receptors may be responsible for the increased bleeding risk with pazopanib treatment.

Pazopanib is known to have an extremely high plasma protein binding compared to other TKIs (24, 48). Therefore, we explored if the presence of blood plasma affected its inhibitory effect on platelet responses. Upon increasing plasma concentrations, it appeared that the inhibition of collagen-induced aggregation became lost at pazopanib concentrations up to 100 μM, whereas the inhibition of PS exposure was still present albeit diminished. Albumin, as a major plasma protein (about 55% of plasma proteins) present at 35–50 mg/mL (49), is considered to be the main pazopanib-binding plasma component (50). This is in agreement with the residual, but consistent inhibition of PS exposure observed in the platelets from patients treated with pazopanib, as well as in whole blood thrombus formation under flow.

Patients with advanced RCC are commonly treated with 800 mg pazopanib per day. The reported steady state, maximal concentration here is 45 μg/mL (51), which corresponds to a concentration of 100 μM. In phase III trials, pazopanib effectively delays disease progression and reduces tumor lesions (25, 52). In spite of the only partial response rate (25), the affinity of pazopanib for VEGF and PDGF receptors is relatively high (42), and likely is higher than that for the intracellular tyrosine kinases implicated in platelet activation. This may explain why the pazopanib dose used for effective cancer treatment does not completely abolish platelet activation processes, but only platelet procoagulant activity (PS exposure), i.e., a response that is most sensitive to inhibition of the cytosolic Ca^2+^ rises.

We and others (24) observed a moderate decrease in platelet count upon pazopanib treatment. This by itself is not expected to result in bleeding, with values still within the normal range of 150–400 × 10^9^ platelets/L (53). This effect may suggest interference in platelet formation (megakaryocytopoiesis). This has not been reported so far, but there is evidence that megakaryocytic signaling via Src family and Syk kinases is required for megakaryocyte migration, and platelet formation (13).

The combination of a reduced platelet count and impaired PS exposure may explain the mostly minor bleeding events observed during pazopanib treatment. In the present study, this held for 3 out of 10 patients, all experiencing epistaxis. This number is relatively high compared to a published clinical trial, reporting mild bleeding in only 13% of the patients (24). However, we like to note that the power of our study is low. Mild bleeding has also been reported with the use of other TKIs in cancer therapy (18, 19). Ibrutinib–affecting collagen- and von Willebrand factor-dependent platelet functions (54)–can cause a risk of mild bleeding in about half of the treated patients, whilst 4–8% of these experiencing major hemorrhages (14). Treatment with the drug ponatinib resulted in a prolonged PFA-100 closure times in patients' blood samples, indicating a loss of platelet function (15). Bleeding occurred here in about 10% of the patients, who however occasionally used other anticoagulants or antiplatelet drugs (55). Treatment with the TKI dasatinib was associated with mild thrombocytopenia and an increased risk of bleeding, likely due to combined effects on megakaryocytes and platelets (56). Also in the latter case, the patients' platelets were less responsive to collagen stimulation, resulting in decreased thrombus formation (57). To take this further, we recently reviewed how distinct TKIs inhibit platelet activation mechanisms, as well as the clinical consequences of antiplatelet effects due to TKI treatment (58). Comparison of affinity profiles of TKIs for platelet targets, as well as literature regarding effects on platelet count, platelet function and bleeding, enabled us to distinguish three categories of TKIs. (i) For several TKIs the bleeding tendency is linked to a lowering of platelet count and/or an impairment of platelet function, (ii) other TKIs are predicated to have an antiplatelet effect, although no bleeding side effects have been reported so far, and (iii) for some TKIs no published data on platelets are available.

In comparison to pazopanib, treatment of RCC patients with sunitinib was found to result in a more profound inhibition of platelet activation (18). Sunitinib is taken up by platelets, and can thus effectively reduce collagen-receptor induced aggregation and thrombus formation (18). A comparative study of sunitinib vs. pazopanib treatment indicated that both drugs provided a progression-free survival benefit when compared to placebo; however, pazopanib had a better safety and quality-of-life profile (52). Sunitinib causes bleeding in up to 20% of patients, and life-threatening bleeding in 3% of the patients (16). As indicated above, for pazopanib these numbers are lower, but still non-negligible (24). Accordingly treatment of metastatic RCC with either pazopanib or sunitinib should be accompanied by special attention of the hemostatic condition, especially when the patients are also treated with antiplatelet drugs.

In summary, the present work demonstrates that platelet treatment with pazopanib *in vitro* results in strong inhibition of collagen-induced platelet activation, aggregation and PS exposure, whereas pazopanib treatment of RCC patients is restricted to inhibition of the platelet procoagulant activity. In combination with the reduction in platelet count, these effects are likely to contribute to the higher bleeding tendency in pazopanib treated RCC patients. Therefore, anti-platelet effects of TKIs should be taken into account in therapy decisions for patients, especially when prescribed in combination with antiplatelet drugs.

## Author contributions

BT performed experiments, analyzed data, and wrote manuscript. MN performed experiments and critically reviewed manuscript. SS performed experiments, wrote medical ethical approval and critically reviewed manuscript. AG provided essential tools, discussed results, and critically reviewed manuscript. MoE discussed results and critically reviewed manuscript. MA included and took care of RCC patients and critically reviewed manuscript. JH designed and discussed experiments, critically reviewed, and revised manuscript. MK designed and performed experiments, discussed results, wrote and revised manuscript.

### Conflict of interest statement

The authors declare that the research was conducted in the absence of any commercial or financial relationships that could be construed as a potential conflict of interest. The reviewer UW declared a past co-authorship with one of the authors JH to the handling Editor.
